# Pistachio green hull and pomegranate peel extracts as two natural antiglycation agents

**DOI:** 10.1002/fsn3.4039

**Published:** 2024-02-20

**Authors:** Mozhgan Roudbari, Mohsen Barzegar, Mohammad Ali Sahari

**Affiliations:** ^1^ Department of Food Science and Technology, Faculty of Agriculture Tarbiat Modares University Tehran Iran

**Keywords:** antiglycation, extract, inhibitor, pistachio, pomegranate

## Abstract

Advanced glycation end products (AGEs) are formed in the final step of the nonenzymatic Maillard reaction, which can contribute to various health problems such as diabetes mellitus, renal failure, and chronic inflammation. Bioactive compounds with antiglycation properties have the potential to inhibit AGE‐related diseases. This study investigated the antiglycation potential of pistachio green hull (PGH) and pomegranate peel (PP) extracts, which are polyphenol‐rich agro‐residues, against fluorescent AGE formation and compared the results with pyridoxine (vitamin B6), metformin, and EDTA (as usual chemical antiglycation agents). The results showed that PGH and PP effectively inhibited the formation of AGEs in bovine serum albumin–glucose (BSA‐Glu) and BSA–fructose (BSA‐Fru) with antiglycation activities ranging from 92% to 97%. PP extract (with an IC_50_ of 94 mg ml^−1^) had a greater antiglycation ability than PGH extract (with an IC_50_ of 142 mg ml^−1^). Also, results indicated that the antiglycation activities of the extracts were comparable to that of pyridoxine, and higher than metformin and EDTA. These findings suggest that the two studied extracts can be used for sustainable production of high‐added‐value food products with a positive effect on consumers' health.

## Graphical abstract

Pistachio green hull (PGH) and pomegranate peel (PP) are widely produced around the world, with a significant portion of them increasingly becoming solid waste, utilizing these peels as natural antiglycation agents in the food industry can be a sustainable and cost‐effective solution. Therefore, in this research, the potential of PGH and PP extracts as natural antiglycation agents for preventing the formation of harmful glycation products in two model systems (BSA‐glucose and BSA‐fructose) are investigated.

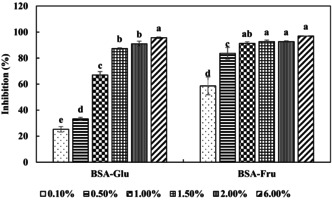

## 1 INTRODUCTION

Glycation is a nonenzymatic reaction in which free amino groups in proteins react with free carbonyl groups of reducing sugars to form a Schiff's base. This is then converted into a more stable product called the Amadori product. Subsequently, advanced glycation end products (AGEs) are generated through a series of oxidation, dehydration, condensation, cross‐linking, and cyclization (González et al., 2020). AGEs are classified as endogenous and exogenous Endogenous AGEs are formed during biological processes in the body, while exogenous ones are often generated during food processing, such as high‐temperature cooking. Furthermore, there is a positive correlation between the protein and fat content of foodstuffs and the production of AGEs (Anwar et al., 2021).

The consumption of foodstuffs containing high amounts of AGEs plays a significant role in their accumulation in the body. The presence of these compounds can have adverse effects on human health and lead to chronic diseases, such as diabetes, atherosclerosis, and Alzheimer's (Henle, 2008). Researchers have reported that certain synthetic compounds or medicines, such as aminoguanidine, metformin, and pyridoxine, can prevent AGEs‐induced diseases, but they may also have some side effects on the body (Anwar et al., 2021; Kazeem et al., 2019).

Currently, many studies are being conducted on the application of natural antiglycation agents obtained from plants. The inhibitory activity of red grape skin extract (RGSE) was tested in the bovine serum albumin–fructose (BSA‐Fru) model system. Results indicated that RGSE has a good antiglycation activity which correlated with the radical scavenging activity of the extract (Jariyapamornkoon et al., 2013). In another study, *Calendula officinalis* L. and *Juglans regia* L. extracts were added into the bovine serum albumin–glucose (BSA‐Glu) reaction mixture, and a good antiglycation activity was observed. These extracts had the same activity as aminoguanidine (Ahmad et al., 2012). Antiglycation activities of 14 plant extracts in the BSA‐Glu and bovine serum albumin–methylglyoxal (BSA‐MGO) model systems were studied in an in vitro assay, and the highest activities were found in star anise, cinnamon, allspice, and cloves, respectively (Starowicz & Zieliński, 2019). Additionally, polyphenolic compounds in hazelnut skin extract were found to inhibit the formation of fluorescent AGEs in the BSA‐MGO model system. The antiglycation activity of hazelnut skin extract was higher than that of aminoguanidine and gallic acid (Spagnuolo et al., 2021). Other natural sources, such as leaf extracts from Chilean bean landraces (Ávila et al., 2022), sorghum bicolor leaf sheath extract (Adetayo et al., 2021), crude and purified extracts of tomato varieties (Błaszczak et al., 2020), barnyard millet phenolics (Anis & Sreerama, 2020), and peanut skin extract (Zhao, Zhu, et al., 2021a), have also been studied for their antiglycation activity by other scientists.

Pistachio (*Pistacia vera* L.) and pomegranate are two popular and widely cultivated fruits in the world, including Iran. In 2020, the total production of pistachio and pomegranate globally was roughly 1,156,832 and 3,846,117 tons, respectively. Since about 40% of the fruits' weight is in their hulls, a large amount of waste is generated annually without being consumed, leading to environmental problems (Aliyari et al., 2020). PGH and PP are rich sources of bioactive compounds, such as phenolic acids (gallic acid, ellagic acid, caffeic acid), and flavonoids (flavonols, e.g., catechin, gallocatechin, epicatechin; anthocyanins, and tannins) (Arjeh et al., 2020; Kaderides et al., 2019). Previous studies have observed that PGH and PP have antioxidative, antimicrobial, antihypertensive, antimutagenic, and antidiabetic properties (Aliyari et al., 2020; Arjeh et al., 2020; Erşan et al., 2016; Kaderides et al., 2019; Ko et al., 2021; Rajaei et al., 2010).

Based on our literature review, there are no published reports on the antiglycation activities of PGH and PP extracts. Also, considering that pistachio and pomegranate peels are widely produced around the world, with a significant portion of them increasingly becoming solid waste, utilizing these peels as natural antiglycation agents in the food industry can be a sustainable and cost‐effective solution. Therefore, we designed this research to investigate the potential of PGH and PP extracts as natural antiglycation agents for preventing the formation of harmful glycation products in two model systems (BSA‐Glu and BSA‐Fru).

## 2 MATERIALS AND METHODS

### 2.1 Materials

D‐glucose, D‐fructose, BSA, EDTA, and sodium azide were purchased from Merck Chemical Co. (Germany). Folin–Ciocalteu, 2, 2‐diphenyl‐1‐ picryl hydrazyl (DPPH) were obtained from Sigma (St. Louis, MO, USA). Pyridoxine hydrochloride (vitamin B_6_) and metformin were provided by Alborz Darou Company (Qazvin, Iran). All of the other chemicals and solvents used in this study were of analytical grade.

### 2.2 Methods

#### 2.2.1 Preparation of PGH and PP extracts

Pomegranate peels (*Malase torshe saveh* variety) and pistachio green hulls (*Ahmad aghaei* variety) were obtained from the Saveh and Yazd Agricultural Research Centers in Iran, respectively. The peels were manually removed, sun‐dried, and maximum drying temperature was 38°C, and ground in a grinder to a 40‐mesh size. Phenolic compounds were extracted from the hulls using distilled water as the solvent, with a liquid‐to‐solid ratio of 15:1, for 8 h at 25°C. The extracts were filtered through Whatman filter paper to remove fine particles. After extraction, the extracts were freeze‐dried and stored at −20°C until further use (Aliyari et al., 2020; Rajaei et al., 2010). Extraction yields of PGH and PP were 14.0% and 12%, respectively.

#### 2.2.2 Determination of total phenolic content

The total phenolic content (TPC) was determined using Folin–Ciocalteu colorimetric method with minor modification (Rajaei et al., 2010). Twenty microliters of each extract was added to 1.4 mL of distilled water and then, 100 μL of Folin–Ciocalteu reagent was added to the mixture. After that, 0.3 mL of Na_2_CO_3_ (7.5%) was added and well mixed. This mixture was incubated for 30 min at 40°C. A ultraviolet–visible spectrophotometer (Agilent Cary 60, USA) was used for measuring the absorbance at 765 nm. Gallic acid was used as a standard for drawing calibration curve. The calibration curve's equation of gallic acid was Y = 0.2725x–0.2554; *r* = 0.9991. Results were expressed as milligrams of gallic acid equivalents per gram dry weight (mg GAE/gdw) (Ghandahari Yazdi et al., 2019).

#### 2.2.3 Antioxidant activity assay

The antioxidant activities of extracts were determined by the DPPH^•^ method (Ghandahari Yazdi et al., 2019) with some modifications. Various concentrations of PGH extract (10.0–200.0 μg mL^−1^, 0.3 mL) and PP extract (10.0–500.0 μg mL^−1^, 0.3 mL) were mixed with 2.7 mL of DPPH^•^ methanolic solution (0.1 mM). The reaction mixture was shaken and incubated for 2 h at room temperature in a dark place. Then, the absorbance was read at 517 nm against a blank. The scavenging activity was calculated using the Equation 1:

(1) $$\text{Free radical scavenging activity}\;\left(\%\right)=\frac{A_{s}-A_{c}}{A_{c}}\times 100$$

where *A*_*c*_ and *A*_*s*_ represent the absorbance of the control and sample. The concentration of the extract which is required to scavenge 50% of the DPPH free radicals was considered as IC_50_.

#### 2.2.4 Glycation reaction of BSA with fructose and glucose

Glycation reactions of BSA with fructose and glucose were done as previously described by Błaszczak et al. (2020), with minor modifications. Briefly, 0.5 mL of BSA (5% w/v) was incubated with 0.5 mL of fructose or glucose (0.8 M) in PBS buffer (0.2 M, pH = 7.4) containing sodium azide (0.002% w/w) in darkness at 37°C for 6 days in the absence or presence of two freeze‐dried extracts (0.1%, 0.5%, 1.0%, 1.5%, 2.0%, and 6.0% w/v) and some reference compounds such as metformin, pyridoxine, and EDTA (100 μL, 1.0 Mm). Test samples were dissolved in PBS (0.2 M, pH = 7.4). The control sample contained all the reaction components without any inhibitor. After incubation, 0.3 mL of the reaction mixture was transferred into a 96‐well ELISA plate to measure the amount of produced fluorescent AGEs using a Cytation three multimode plate reader (BioTek Instruments, Winooski, VT, USA) by measuring fluorescence intensity at excitation and emission wavelengths of 340 and 435 nm, respectively. The inhibitory activity was evaluated by Equation 2:

(2) $$\text{Inhibition}\;\left(\%\right)=\left[1–\left(\frac{F_{s}}{F_{c}}\right)\right]\times 100$$

where *F*_*s*_ and *F*_*c*_ are the fluorescence intensities of the test sample and control, respectively.

#### 2.2.5 Statistical analysis

All analyses were performed in triplicate, and the results were reported as mean ± standard deviation. Mean comparisons were performed using the one‐way ANOVA method, and a *p*‐value lower than 0.05 was considered statistically significant. Statistical analysis was performed using SAS software (version 13).

## 3 RESULTS AND DISCUSSION

### 3.1 Total phenolic content (TPC) of studied extracts

Results showed that TPC of PGH and PP extracts was 810.0 ± 2.1 and 861.0 ± 2.6 mg GAE/gdw, respectively. According to the literature, phenolic compounds included in PGH can be categorized into three major groups; phenolic acids, flavonoids, and tannins (Arjeh et al., 2020; Erşan et al., 2016). Ghandahari Yazdi et al. (2019) reported that the main phenolic compounds of PGH extract were gallic acid and phloroglucinol. Phenolic compounds of PP have been presented in previous reports; tannins (ellaganitans, gallotannins, gallagyl esters), flavonoids, and phenolic acids (hydroxycinnamic and hydroxybenzoic acids) (Aliyari et al., 2020; Kaderides et al., 2019).

### 3.2 Determination of antioxidant activity

Results of DPPH^•^ free radical assay showed that PGH and PP extracts have significant radical scavenging activity with IC_50_ of 142.01 ± 1.0 μg mL^−1^ and 94.0 ± 1.6 μg mL^−1^, respectively. By comparing the results, it can be found that the antioxidant activity of PP extract is higher than PGH, but both extracts are a rich source of bioactive compounds compared to other plant sources (Table 1).

**TABLE 1:** Total phenolic content and antioxidant activities of some plant extracts.

Extract	Total phenolic content	Antioxidant capacity (method)	References
Extract of *P. edulis* seeds	227 mg GAE/gdw	20.4 μg/mL (DPPH^•^)	Dos Santos et al. (2022)
Hazelnut skin extract	70 mg GAE/gfw	0.28 mmol TE/gfw (TEAC and ORAC)	Spagnuolo et al. (2021)
*Sorghum bicolor* leaf sheath extract	11.68 mg GAE/gdw	33.4 mg/mL (DPPH^•^)	Adetayo et al. (2021)
Green kiwifruit peel extract	51.2 mg GAE/gdw	195.1 mM TE/gdw (DPPH^•^)	Guthrie et al. (2020)
*Stevia rebaudiana* Bertoni extract	20.1 mg GAE/g	10 mg/mL (DPPH^•^)	Escutia‐López et al. (2020)
Green pepper extract	24.9 mg GAE/mL	3.1 mg GAE/mL (DPPH^•^)	Favre et al. (2020)
*Opuntia macrorhiza* fruit skin	1.55 mg/g dry extract	0.99 mg/mL (DPPH^•^)	Chahdoura et al. (2019)
*Opuntia microdasys* fruit skin	0.58 mg/g of dry extract	1.34 mg/mL (DPPH^•^)	
Calendula	0.67 g GAEs/L of infusion	177mmole TEs/L of infusion (ORAC)	Ho et al. (2014)
Chamomile	0.53 g GAEs/L of infusion	298 mmole TEs/L of infusion (ORAC)	
Chrysanthemum	0.50 g GAEs/L of infusion	209 mmole TEs/L of infusion (ORAC)	
Cornflower	0.62 g GAEs/L of infusion	192 mmole TEs/L of infusion (ORAC)	
Jasmine	0.73 g GAEs/L of infusion	268 mmole TEs/L of infusion (ORAC)	
Lavender	0.78 g GAEs/L of infusion	276 mmole TEs/L of infusion (ORAC)	
Neroli	0.86 g GAEs/L of infusion	352 mmole TEs/L of infusion (ORAC)	
Rose	2.75 g GAEs/L of infusion	422 mmole TEs/L of infusion (ORAC)	
Sweet Osmanthus	2.13 g GAEs/L of infusion	634 mmole TEs/L of infusion (ORAC)	
Pistachio green hull and pomegranate peel extracts	810.0 mg GAE/gdw 861.0 mg GAE/gdw	142.01 μg/mL and 94.0 μg/mL (DPPH^•^)	This work

Abbreviations: AAE, milligrams of ascorbic acid equivalents; DPPH, 2, 2′‐diphenyl‐1‐picrylhydrazyl; dw, dry weight; GAE, gallic acid equivalents; ORAC, Oxygen radical antioxidant capacity; TAC, Total antioxidant capacity; TE, trolox equivalent; TPC, total phenolic content.

### 3.3 Antiglycation activity

**FIGURE 1:**
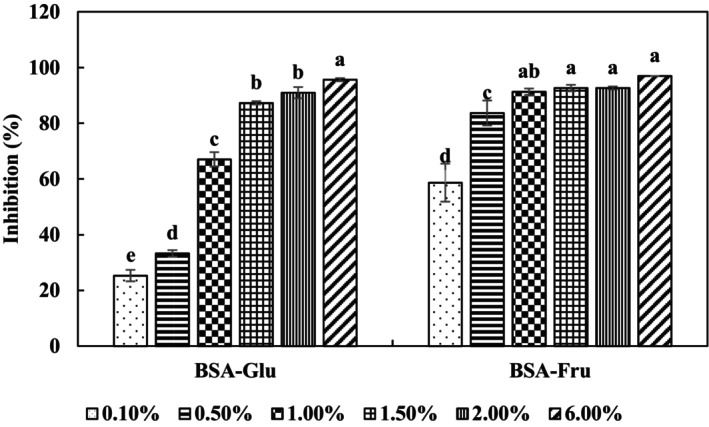
Antiglycation activity of pomegranate peel (PP) extract (6.0% w/v) in the two model systems. Results are presented as mean ± s.d. (*n* = 3). BSA‐Glu (bovine serum albumin–glucose); BSA‐Fru (bovine serum albumin–fructose); Different letters on the columns indicate significant differences (*p* < 0.05); Concentration of pyridoxine was 1.0 mM.

As shown in Figure 1, the PP extract inhibited the glycation reaction in a concentration‐dependent manner. The percentage of inhibition in the BSA‐Glu system was slightly less than 25.5% initially, but it increased significantly and reached to 95.7% at the highest concentration (6.0% w/v, *p* < 0.05).

In the BSA‐Fru system, the PP extract's antiglycation activity was about 58.66% at 0.1% w/v, and it reached ~92.7% and then remained constant up to 2.0% w/v. The highest activity (97%) was observed at 6.0% w/v of PP, which was significantly higher than the other concentrations (*p* < 0.05).

**FIGURE 2:**
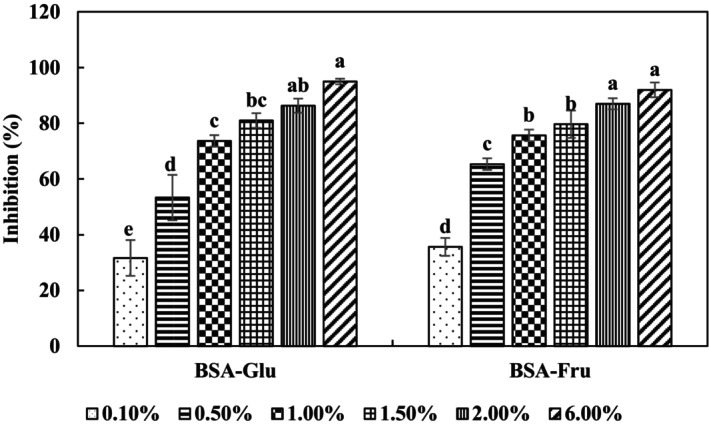
Antiglycation activity of pistachio green hull (PGH) extract (6.0% w/v) in the two model systems. Results are presented as mean ± s.d. (*n* = 3). For abbreviations and other conditions see Figure 1 legend.

As can be seen in Figure 2, there is a direct correlation between the concentration of the PGH extract and its antiglycation activity. The results of the BSA‐Glu system revealed that the initial antiglycation activity was 31.7% (at 0.1% w/v of extract) and increased to 95% at 6.0% w/v. In addition, the PGH extract showed a similar behavior in the BSA‐Fru model system, with an initial antiglycation activity of 35.66% (at 0.1 w/v %) that reached to 92% at 6.0% w/v.

**FIGURE 3:**
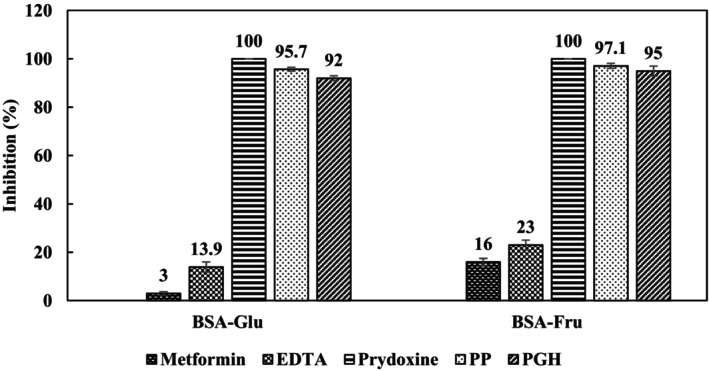
Antiglycation activities of PGH and PP extracts (6.0% w/v) compared to metformin and pyridoxine, EDTA in two model systems. Results are presented as mean ± s.d. (*n* = 3). For abbreviations and other conditions, see Figure 1 legend. Concentration of metformin, pyridoxine, and EDTA was 1.0 mM.

In this research, PP and PGH extracts exhibited different antiglycation activities. In this research, PPE and PGHE exhibited different antiglycation activities. The PPE exhibited greater activity in comparison with the PGHE. This difference in activity could be attributed to the distinct profile and quantity of phenolic compounds found in each extract. Pomegranate peel is rich in ellagitannins (Saroj et al., 2020), while pistachio hull mainly contains proanthocyanidins (Mandalari et al., 2021). Also, pomegranate peel has higher levels of flavonoids, as well as anthocyanins compared to pistachio hull (Zhao, Shen, et al., 2021b). To confirm the potential inhibitory effect of PGH and PP extracts (at 6.0% w/v), their activities were evaluated and compared with two common inhibitors (e.g., metformin and pyridoxine) and a chelator (EDTA) (at 1.0 mM). As shown in Figure 3, pyridoxine was able to completely inhibit the glycation reaction. Schalkwijk and Miyata (2012) reported that pyridoxine reacts with the produced active carbonyl compounds and oxygen free radicals during the glycation process. In contrast, metformin did not significantly affect the glycation process in the BSA‐Glu system but had a slightly inhibitory effect on the BSA‐Fru system. This result is in agreement with the results reported by Sadowska‐Bartosz et al. (2014), who found that metformin slightly decreases the rate of glycoxidation in systems containing glucose, but has a moderate inhibitory effect on the glycoxidation in the presence of fructose.

EDTA did not show a significant effect on the two studied systems. Interestingly, the antiglycation activity of the pomegranate peel and pistachio green hull were comparable to vitamin B_6_ and significantly higher than EDTA and metformin in both systems.

It seems that the antiglycation activities of the two studied extracts are attributed to their high contents of phenolic compounds. Phenolic compounds are believed to inhibit glycation and its propagation through various mechanisms, including reducing oxidative pathways by scavenging free radicals, blocking reactive carbonyl groups that can react with different biomolecules, leading to oxidative stress, and chelating metal ions (Khan et al., 2020). Muthenna et al. (2012) indicated that ellagic acid can inhibit the formation of fluorescent and nonfluorescent AGEs by blocking active carbonyl groups. Umadevi et al. (2014) reported that gallic acid scavenges reactive carbonyls involved in the formation of AGEs. In Yin et al.'s study (2014), epigallocatechin gallate, epigallocatechin, epicatechin gallate, and epicatechin were found to donate electrons or hydrogen to neutralize radicals or oxidants. Consequently, these compounds are likely to contribute to antiglycation activity through their radical‐scavenging capacities. Previous studies have investigated the antiglycation activity of anthocyanins such as delphinidin‐3‐rutinoside and cyanidin‐3‐O‐rutinoside, which can trap reactive carbonyl species and prevent their reaction with other molecules (Chen et al., 2014; Thilavech et al., 2015). Li et al. (2014) have reported that quercetin can suppress α‐dicarbonyl compounds that induce protein glycation and trap methylglyoxal. Another study reported that epicatechin, p‐coumaric acid, and gallic acid are able to decrease protein carbonyl, thiol oxidation, and fluorescence AGE formation (Khan et al., 2020). Moreover, gallic acid has been found to reduce the levels of oxidative stress markers (Gao et al., 2019). Another study found that phloroglucinol inhibits the formation of AGEs due to its antioxidant activity (Drygalski et al., 2021). Many studies have reported that ellagitannins, gallotannins, and gallagyl esters in the PP extract (Singh et al., 2018) and gallic acid, quercetin, phloroglucinol, theogallin, galloyl derivatives, catechin, and pyrogallol in the PGH extract (Arjeh et al., 2020) are responsible for their antioxidant activity. Atta et al., 2023 reported that pistachio extract (at a dose of 1 mg/mL) showed the highest inhibitory activity on the formation of dicarbonyl chemicals in BSA glycation processes compared to other studied nuts. Therefore, the present data are consistent with the literature regarding the antiglycation mechanism of phenolic compounds.

Table 2 presents a selection of natural inhibitors whose inhibitory effects can be compared to those of the studied extracts. The use of these extracts is significant, as they not only reduce waste and promote resource efficiency but also offer significant potential as functional ingredients for controlling AGEs in food processing.

**TABLE 2:** Comparison of antiglycation activities of our studied extracts with some recent reports.

Model system	Inhibitor	Function	References
BSA (50 mg/mL), fructose or glucose (0.8 M)	PGH and PP extracts	The best inhibitory activities of PGH and PP extracts (at 6.0%) were ~95.0 (BSA‐Glu system) and 97.0% (BSA‐Fru system), respectively	Present study
BSA (10 mg/mL), glucose (0.5 M)	Leaf extracts from Chilean bean landraces	IC_50_ values of antiglycation activity of Coscorrón, Frutilla, Magnum, Peumo, Sapito, and Tórtola bean leaf extracts were 336, 681.9, 585.8, 462.7, 326.3, and 373.3 μg/mL, respectively.	Ávila et al. (2022)
BSA (10 mg/mL), glucose (0.8 M)	Peanut skin extract	At the concentration of 0.2 mg/mL, the inhibition activity was about 75%.	Zhao, Zhu, et al. (2021a)
BSA (10 mg/mL), Fructose (500 mM)	Barnyard millet (*Echinochloa frumentacea*)	A 73% reduction in AGE formation was observed at a concentration of 100 μg/mL.	Anis and Sreerama (2020)
BSA (10 mg/mL), glucose (500 mM)	The ginger methanolic extract	The ginger methanolic extract (600 μg/mL) showed 44.50% glycation as compared to glycated BSA incubated without ginger extract (100%).	Anwar et al. (2020)
BSA (10 mg/mL)‐ Glucose (1.0 M) BSA (1 mg/mL)‐MGO (5 mM)	Crude and purified extracts of different tomato varieties	IC_50_ values of antiglycation activity in purified extracts of black prince tomatoes that were the most potent inhibitors of AGEs in BSA‐Glu (7.20 mg/mL) and BSA‐MGO (9.53 mg/mL) models.	Błaszczak et al. (2020)
BSA (5 mL, 10 mg/mL)‐glucose (5 mL, 500 mM)	*Opuntia macrorhiza* fruit peel polysaccharides (2.5 mL, 10 mg/mL)	The best antiglycation activity was 71.5%	Amamou et al. (2020)

### 3.4 Antiglycation activity of the mixed extracts

**FIGURE 4:**
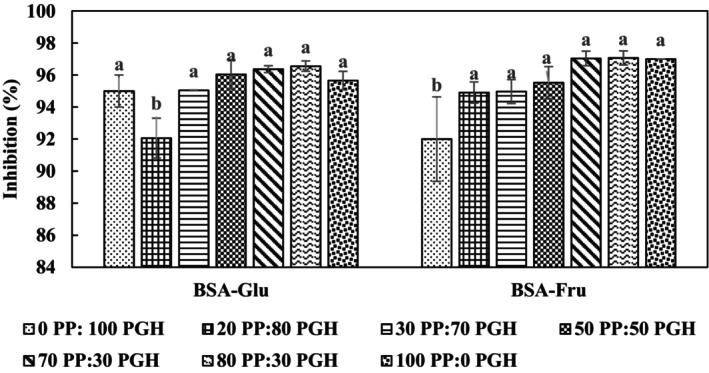
Effect of different ratios of PP: PGH extracts (6.0% w/v) on inhibition of glycation reaction. Data are means ± s.d. (*n* = 3). For abbreviations and other conditions, see Figure 1 legend.

According to the results presented in Figure 4, the mixture of extracts increased the antiglycation activity up to 97%. In the BSA‐Fru system, the lowest activity was observed for the 0:100 ratio (PPE:PGHE), and no significant difference was found among the other ratios (*p* < 0.05). Similar results were obtained in the BSA‐Glu system, where increasing ratios of PPE to PGHE resulted in higher antiglycation activity. However, the lowest antiglycation activity was observed at the 20:80 ratio of PPE:PGHE. These findings are consistent with previous studies that have reported higher TPC and antioxidant activity in PP extract compared to PGH extract (Aliyari et al., 2020).

It has been recently reported that mixing different phenolic compounds can exhibit synergistic effects in biochemical processes (Spagnuolo et al., 2021). For instance, combining gallic acid with caffeic acid, as well as quercetin, gallic acid, caffeic acid, quercetin, gallic acid, and rutin with each other demonstrated high synergistic effects (antioxidant activity). Therefore, the antioxidant and antiglycation properties of plant extracts could be explained by the synergistic effect of phenolic compounds present in the extracts (Ramkissoon et al., 2013). Additionally, from an economic standpoint, mixing compounds may prove cost‐effective since less material will be consumed.

## 4 CONCLUSION

In this study, the antiglycation activities of two natural agricultural wastes, PGH and PP extracts, were investigated in two model systems. The results indicated that the studied extracts, either individually or in combination, could significantly inhibit the formation of fluorescent AGEs, with activities comparable to that of vitamin B_6_. Furthermore, the PP extract exhibited higher antiglycation activity than the PGH extract, likely due to its higher total phenolic content and antioxidant activity. Therefore, these extracts may have the potential for use as a rich source of bioactive molecules in food products that are susceptible to the Maillard reaction (such as cookies, biscuits, bread, and coffee).

## AUTHOR CONTRIBUTIONS

**Mozhgan Roudbari:** Data curation (equal); formal analysis (equal); writing – original draft (equal). **Mohsen Barzegar:** Conceptualization (equal); project administration (equal); supervision (equal); writing – review and editing (equal). **Mohammad Ali Sahari:** Formal analysis (equal); validation (equal); writing – review and editing (equal).

## CONFLICT OF INTEREST STATEMENT

There is no conflict of interest in this paper.

## ETHICS STATEMENT

On behalf of all coauthors, I, Dr. Mohsen Barzegar, declare that this article has not been published in or is not under consideration for publication elsewhere. All authors were actively involved in the work leading to the manuscript and will hold themselves jointly and individually responsible for its content. Human or animal testing is unnecessary in our study.

## Data Availability

The data that support the findings of this study are available on request from the corresponding author.
